# Distribution, Cleavage and Lipidation of Atg8 Fusion Proteins in *Spodoptera litura* Sl-HP Cells

**DOI:** 10.1371/journal.pone.0096059

**Published:** 2014-05-02

**Authors:** Xiaojuan Zhang, Hongjuan Lu, Hui Ai, Rong Peng, Yongbo Yang, Aiying Li, Huazhu Hong, Jianxin Peng, Kaiyu Liu

**Affiliations:** School of Life Sciences, Central China Normal University, Wuhan, China; Wuhan Bioengineering Institute, China

## Abstract

Atg8 proteins fused with tags are commonly used to detect autophagy. The expression patterns of Lepidopteran insect *Atg8* are relatively well documented. However, the influence of protein tags on characterization of Atg8 is still not very clear. Our results showed that endogenous *Spodoptera litura* Atg8 and HA tagged Atg8 driven by the baculovirus ie2 promoter were enriched in cytoplasm. The recombinant plasmid pEGFP-Atg8(EGFP) in which *Atg8* contained a stop codon was constructed and expressed. Green fluorescence was accumulated in cytoplasm. However, red fluorescence was located in both cytoplasm and nucleoplasm in most cells transfected with the recombinant plasmid pmCherry-Atg8(EGFP). In contrast to pEGFP-Atg8(EGFP), green fluorescence was also located in both cytoplasm and nucleoplasm in most cells transfected with the recombinant plasmid pie2/EGFP-Atg8 driven by the baculovirus ie2 promoter in which the CMV promoter and EGFP nucleotide sequences were removed, and the high level of the EGFP-Atg8 expression significantly increased its abundance in nucleoplasm. HA-Atg8 expressed at high level through baculovirus under the control of polyherin promoter was also localized in cytoplasm and nucleoplasm. The cleavage of mCherry-Atg8 was different from that of EGFP-Atg8. Both the mutant mCherry-Atg8^F77/79A^ resulting in non-cleavage of the Atg8 and the mutant mCherry-Atg8^G^ exposing its glycine residue at the end of C-terminus were also localized in cytoplasm and nucleoplasm. The increase of autophagosomes decreased the abundance of mCherry-Atg8 in nucleoplasm. In addition, the ratio of HA-Atg8-PE/HA-Atg8 was less than that of endogenous Atg8-PE/Atg8. These results demonstrated that the Atg8 is located in both nucleus and cytoplasm when expressed at high level and exported to the cytoplasm when autophagy is activated, and the fusion tags of Atg8 might have influence on the processing of Atg8 fusion proteins.

## Introduction

Autophagy (macroautophay) is an intracellular bulk degradation pathway through the lysosomal machinery in eukaryotes [1], [2]. By removing excessive, unused or damaged cytoplasmic components and organells, autophagy serves to maintain intracellular homeostasis [3]. In multicellular organisms, autophagy is involved in diverse physiological processes, including development, immunity, and protection under pressure and tumor suppression [4]–[6].

Autophagy can be divided into three stages: Induction of autophagy, autophagosome formation and the fusion of autophagosome with lysosome [7], [8]. Studies in both yeast and mammalian systems have demonstrated that Atg8 plays a dual role in the autophagosome formation process, coupling between selective incorporation of autophagy cargo and promoting autophagosome membrane expansion and closure [9], [10]. It is well known that Atg4 cleaves the carboxyl terminus of Atg8 homologues and leaves glycine residue at the C-terminus of Atg8 [11]. Following this process, Atg8 is conjugated to phosphatidylethanolamine (PE) to form Atg8-PE which binds to membrane structure [11], [12]. The reaction in vivo is catalyzed by the sequential actions of Atg7 (E1-like activating enzyme), Atg3 (E2-like conjugation enzyme) and the Atg12−Atg5-Atg16 complex (E3-like ligase) [13]. In addition, the deconjugation activity of Atg4 on Atg8-PE is also required for normal autophagy [13]–[15]. This proteolytic activity is diminished by N-ethylmaleimide, an inhibitor of cystein protease including yeast Apg4/Aut2 [16].

Fluorescent proteins are widely used to track localization and transportation of target protein [17], [18]. Although LC3/Atg8 is currently thought to function primarily in the cytosol, the site of autophagosome formation, many studies have reported that EGFP-LC3/Atg8 is enriched in nucleoplasm rather than in cytoplasm [18]–[21]. Nucleocytoplasmic distribution and dynamics of the autophagosome marker EGFP-LC3 have also been investigated in mammalian cells [22]. However, in Lepidoptera, little is known about the function and action mechanism of Atg8 that has been primarily characterized [23], [24]. In the present study, the Atg8 protein was fused with different tags and expressed in insect cells, and their localization, shuttling between cytoplasm and nucleus, degradation and lipidation were investigated.

## Materials and Methods

### Cell Culture and Reagents

*Spodoptera litura* Sl-HP cells stored in our laboratory were grown in Grace^,^s insect medium supplemented with 10% fetal bovine serum (Life Technologies Corporation) at 28°C [23]. Mouse anti-mCherry polyclonal antibody, mouse anti-EGFP monoclonal antibody, mouse anti-HA antibody and Dylight 488-conjugated goat anti-mouse IgG were purchased from the company (Earthox, LLC, San Francisco, CA, USA). Mouse anti-Atg8 polyclonal serum was prepared using *Helicoverpa armigera* Atg8 (GenBank accession number: JQ739159) as antigen in our laboratory [23].

### Construction of Plasmids and Transfection

**Figure 1:**
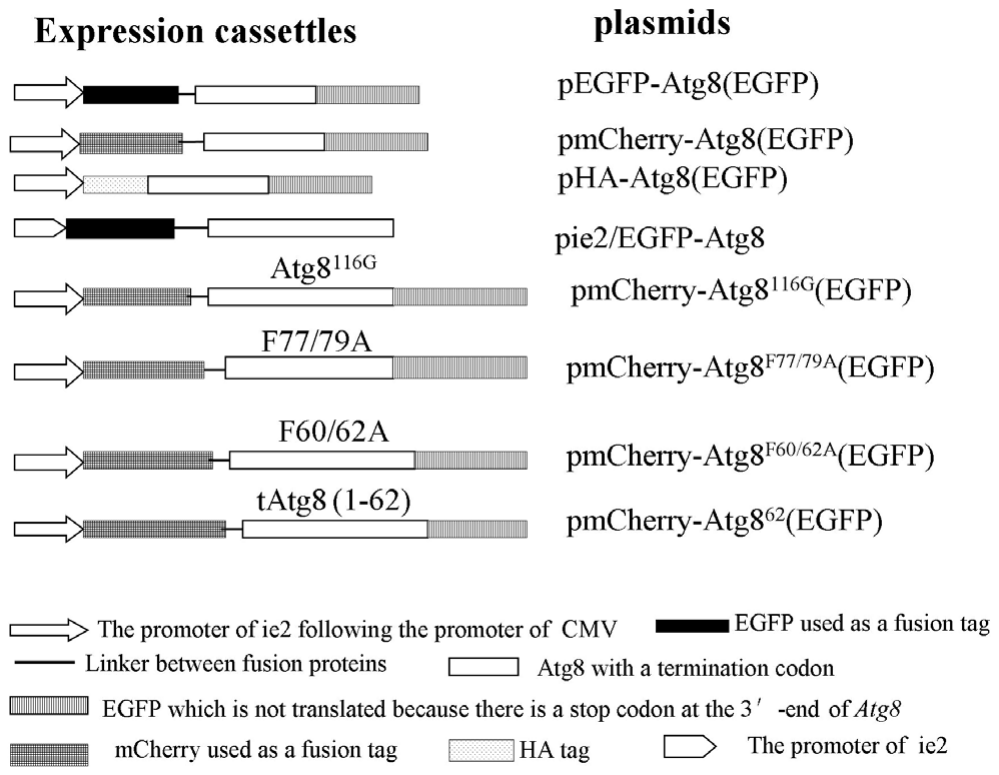
Construction of plasmids. The commercial plasmids pEGFP-C1 and pEGFP-N1 contain the CMV promoter which acts in mammalian cells, and the promoter of baculovirus ie2 which works in insect cells derives from the plasmid pIZ-V5/His. All the recombinant plasmids contained the promoter of ie2 following the promoter of CMV except pie2/EGFP-Atg8, which had no the promoter of CMV. The EGFP after Atg8 did not express because Atg8 has a stop codon. PCR was used for site-directed mutagenesis and truncation of open reading frames. Atg8^116G^ has a glycine (G) residue at C terminus (the 116^th^ amino acid residue). Atg8^62^ had 62 amino acid residues of the N terminus of Atg8. F77/79A and F60/62A indicated that tyrosine (F) residues were replaced with alanine (A) residues.

*H. armigera* Atg8 was amplified using cDNA prepared from mRNA of larvae as template by PCR. The baculovirus ie2 promoter was amplified from plasmid pIZ/V5-His (Life Technologies Corporation) in order to express Atg8 fusion proteins in insect cells. Firstly, the ie2 promotor was inserted after the promoter of CMV of the plasmid pEGFP-N1 (Clonetech, CA). Then the EGFP or mCherry gene without a stop codon was inserted into multi-cloning sites. Following this step, Atg8 was inserted after EGFP or mCherry by a linker of 6 glycine residues in the same reading frame with fluorescent proteins to produce the plasmids pEGFP-Atg8(EGFP) and pmCherry-Atg8(EGFP), respectively. The plasmid pHA-Atg8(EGFP) was obtained by replacing *EGFP-Atg8* with *HA-Atg8.* The plasmid pie2/EGFP-Atg8 was constructed as following: the CMV promoter of the plasmid pEGFP-C1 (Clonetech, CA) was replaced with the ie2 promoter of baculovirus, then Atg8 was inserted after EGFP by a linker of glycine residues in the same reading frame with EGFP. Based on the plasmid pmCherry-Atg8(EGFP), Atg8 was truncated at the C-terminus (deletion from the 63th amino acid residue (Atg8^62^)or the last one (Atg8^116G^), or mutated at the specific sites (F60/62A and F77/79A). The construction scheme of recombinant plasmids was shown in Figure 1 and the main primers used in this study were listed in Table 1.

**Table 1:** The primers used in the study.

Fragments for expression	Names of primers	Sequences of primers
HA-Atg8	HAtg8-FP	5-GGGGTACCGGCCACCATGTACCCATACGATGTTCCGGATTACGCTATGAAATTCCAATAT-3
	HAtg8-RP	5-CGGGATCCTTAATATCCATATACATTCT-3
EGFP-Atg8	EAtg8-FP	5-CCCAAGCTTCGGGCGGTGGAGGGATGAAATTCCAATATAAAGAAG-3
	EAtg8-RP	5-CGGGATCCTTAATATCCATATACATTCT-3
mCherry-Atg8	mAtg8-FP	5-GGGGTACCGATGAAATTCCAATATAAAGAAG-3
	mAtg8-RP	5-CGGGATCCTTAATATCCATATACATTCT-3

Sl-HP cells were seeded onto sterile microscope coverslips (13-mm diameter) in a 24-well tissue culture plate over night. A transfection mixture was prepared by mixing plasmid DNA (1 µg) and cellfectin reagent (2 µl) for each well according to the instruction supplied by the company (Life Technologies Corporation) and incubated for 30 min at room temperature before use. Then the medium was removed from the wells, the cells were washed twice with serum-free medium, and the transfection mixture supplemented with serum-free medium was added into the wells gently. The cells were then incubated with the mixture for 5 h at 28°C. After the indicated incubation time, the cells were washed twice with serum-free medium. Finally, the cells were incubated with Grace^,^s insect medium supplemented with 10% FBS and observed under a fluorescence microscope or laser scanning focal microscope and photographed 24 or 36 h post transfection.

### Construction of Recombinant Bacmid

The *HA-Atg8* was amplified by PCR using a pair of primers (HA-Atg8-FP and HA-Atg8-RP) and the pEGFP-Atg8(EGFP)was used as a template. The forward primer HA-Atg8-FP, 5'- CGGGATCCGCCACCATGTACCCATACGATGTTCCGGATTACGCTATGAAATTCCAATAT-3', contained sequences for a BamH I site and HA tag before the start codon of the *Atg8* gene and the reverse primer HA-Atg8-RP, 5'- CCAAGCTTTTAATATCCATATACATTCT-3' included a Hind III site and a stop codon. The amplified product was subcloned into the corresponding sites in pFastBac Dual plasmid (Life Technologies Corporation) under the control of the baculovirus polyhedrin promoter. The purified plasmid DNA was transformed into DH10Bac *E. coli* (Life Technologies Corporation) which contained a baculovirus shuttle vector to obtain the recombinant Bacmid-HA-Atg8. The Bacmid DNA was transfected into Sf9 cells to generate the recombinant baculovirus (rBacHA-Atg8) which was used to infect Sl-HP cells at a moi of 0.2. The localization of HA-Atg8 in the infected cells were evaluated by immunofluroescence assay using anti-HA antibody according to the standard protocol.

### Cell Staining for Immunofluorescence

Sl-HP cells were seeded onto sterile microscope cover slips (13-mm diameter) in a 24-well tissue culture plate. The cells were then transfected with plasmids as described above and cultured for 24 h at 28°C. After the indicated incubation time, the cells were washed twice with phosphate-buffered saline (PBS) (1 mM Na_2_HPO_4_, 10.5 mM KH_2_PO_4_, 140 mM NaCl, 40 mM KCl, pH 7.4) and then fixed with 4% paraformaldehyde. After washing five times, the cells were blocked with 2% BSA in PBS for 2 h at room temperature. Then the fixed cells were incubated with primary antibody at room temperature for 1 h. After three times of washing with PBS, the cells were incubated with fluorescent dye-label goat anti-mouse immunoglobulin G (IgG) secondary antibody (1∶1000 diluted in PBS containing1% BSA) for 30 min at room temperature. Finally, the cells were washed three times with 1×PBS, mounted in buffered glycerol under a cover slip and observed under a fluorescence microscope or laser scanning focal microscope, and photographed.

### Western Blot Assays

Sl-HP cells were transfected with each recombinant plasmid as described above. After 36 h post transfection, the cells were collected, washed with PBS containing protease inhibitor and suspended in SDS-PAGE sample buffer. The samples were then separated on a SDS-10% polyacrylamide gel. After electrophoresis, proteins were electrotransferred onto a PVDF membrane. The membrane was blocked with 5% nonfat milk in TBS-T buffer for 2 h at room temperature, and then incubated with primary antibody. Following three times of washing with TBS-T, the membrane was incubated with fluorescent secondary antibody at a 1∶5000 dilution. Finally, the membrane was washed for three times with TBS-T, and then bands were visualized and photographed using the Odyssey system (LI-COR Biosciences Company).

## Results

### The Differential Distribution of Atg8 Fusion Proteins

**Figure 2:**
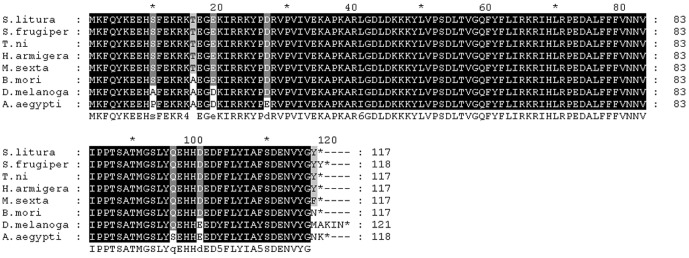
A multiple alignment of amino acid sequences of Atg8 from different insect species. *S. litura SlAtg8* (GenBank accession number: JX183217), *Spodoptera frugiperda SfAtg8* (Spodobase: Sf2M09420-5-1), *Trichonplusia ni TnAtg8* (GenBank accession number: JX183216), *Helicoverpa armigera HaAtg8* (GenBank accession number: JQ739159), *Manduca sexta MsAtg8* (Manduca Base: CUFF.28656.1), *Bombyx mori BmAtg8* (GenBank accession number: FJ416330.1), *Drosophila melanogaster DmAtg8* (GenBank accession number: NM167245.2), *Aedes aegypti* gaba(a) receptor (GenBank accession number: AY736002.1).

The amino acid sequences of Atg8 among insects are highly conserved, and the amino acid sequences of the Atg8 of the *S. Litura* and the *H. armigera* are identical although they are from different genera of Lepidopteran insect (Figure 2). Thus, a series of plasmids expressing Atg8 fusion proteins were constructed using *H. armigera Atg8* gene in the present study.

**Figure 3:**
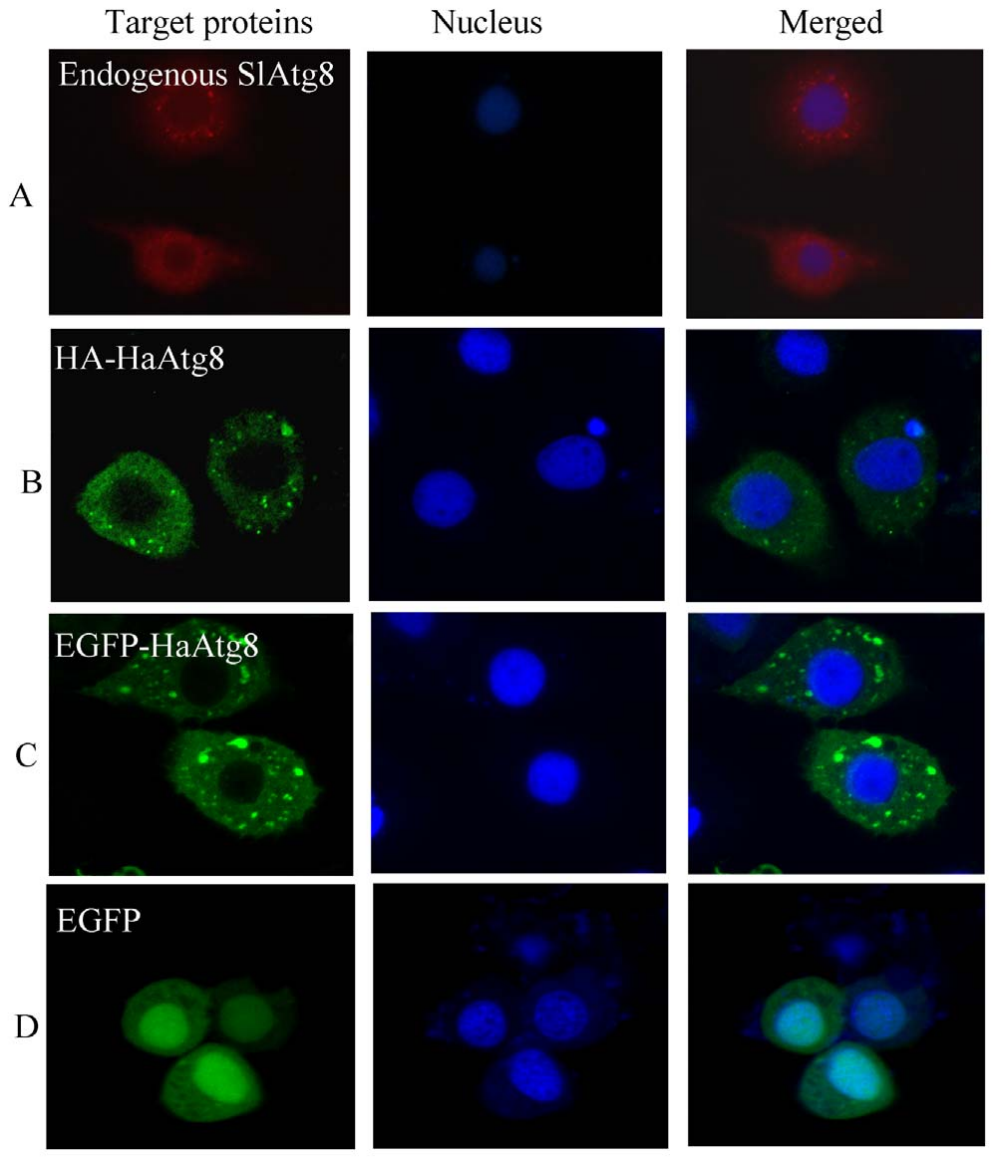
Subcellular distributions of endogenous Atg8 and Atg8 fusion proteins. (A)endogenous Atg8; (B) pHA-Atg8(EGFP), the EGFP of which in brackets is non coding extra-sequences; (C) pEGFP- Atg8(EGFP); (D) pEGFP.

**Figure 4:**
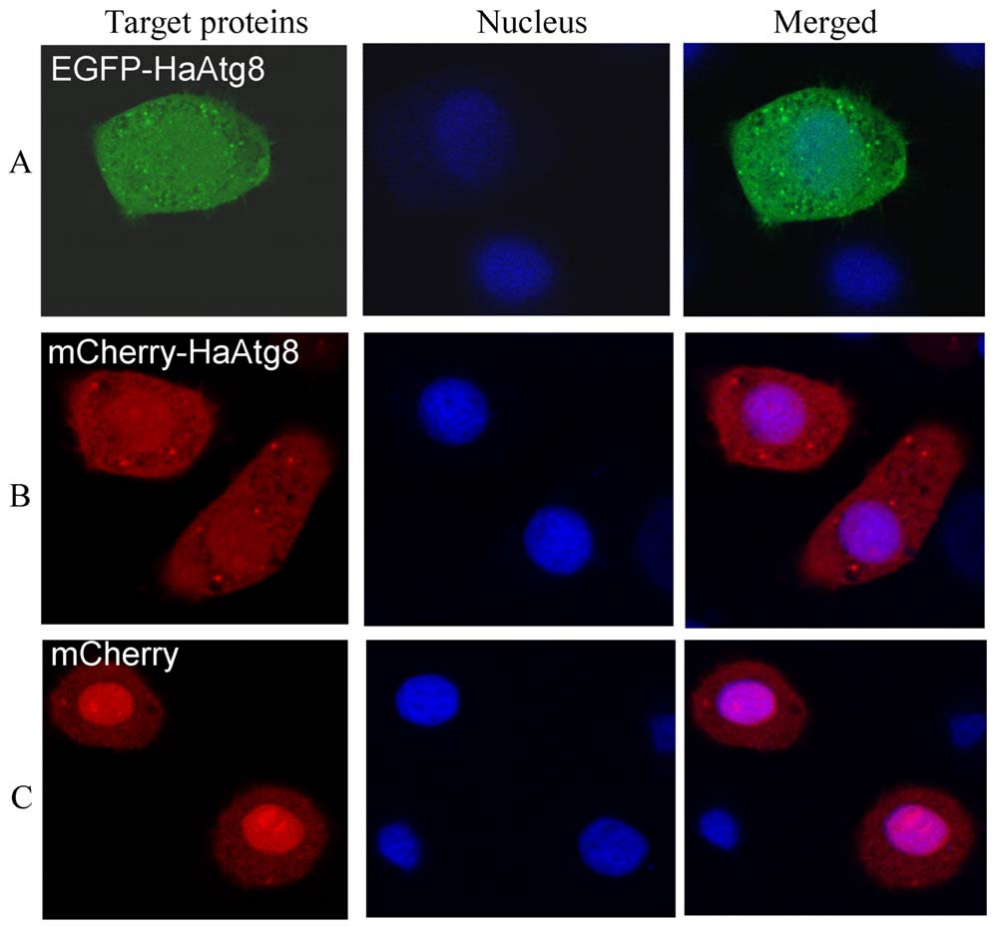
Atg8 fusion proteins located in both cytoplasm and nucleoplasm. (A) pie2/EGFP-Atg8; (B) pmCherry-Atg8(EGFP); (C) pmCherry(EGFP).

To determine the localization of Atg8 in Sl-HP cells, the distribution of endogenous Atg8, HA-Atg8 and fluorescent protein tagged Atg8 were observed. Immunofluorescence cell staining revealed that endogenous *S. lituras* Atg8 was enriched in cytoplasm of Sl-HP cells (Figure 3 A). The same result was observed in the recombinant plasmid pHA-Atg8(EGFP) transfercted cells in which the *HA* tagged *Atg8* was under the control of the baculovirus ie2 promoter (Figure 3B). To confirm this result, the recombinant plasmid pEGFP-Atg8(EGFP) was constructed and expressed in Sl-HP cells. Both fluorescence microscope and laser scanning confocal microscope revealed an accumulated green fluorescence in cytoplasm (Figure 3C). Interestingly, when the plasmid pie2/EGFP-Atg8 or the plasmid pmCherry-Atg8(EGFP) was expressed in Sl-HP cells, the green fluorescence or the red fluorescence was localized in both cytoplasm and nucleoplasm (Figure 4A and B), indicating that the subcellular location of the Atg8 was greatly affected by the fluorescent protein tags.

### The Different Localization of the Atg8 Protein Was Associated with Its Expression Level

**Figure 5:**
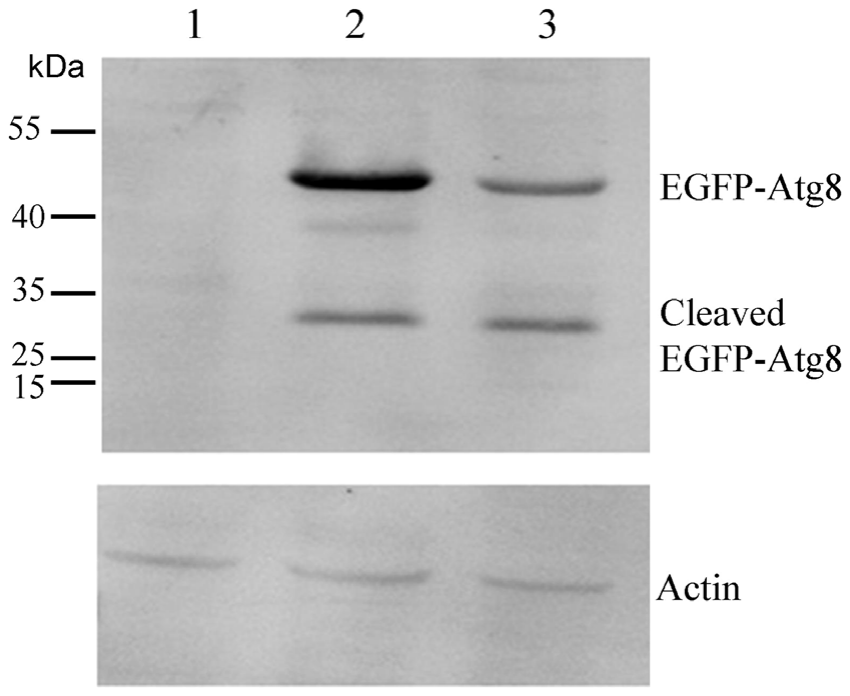
The expression level of EGFP-Atg8 in the cells transfected with the different plasmids. Lane 1. control; lane 2. pie2/EGFP-Atg8; lane 3. pEGFP-Atg8(EGFP). Mouse anti-GFP monoclonal antibody and anti-tubulin antibody were used as primary antibodies for western blot assay.

To determine whether the alteration of the localization of the Atg8 proteins was associated with the specific protein(s) expressed via different plasmids, western blot assay was used to detect the expression level of proteins. The result demonstrated that the size of EGFP-Atg8 fusion protein with different distribution in Sl-HP cells was identical (Figure 5). However, their expression levels differed significantly (Figure 5) although no significant difference of the transfection efficiency was observed between the two plasmids. The fusion protein was located in both cytoplasm and nucleus when it was expressed at high level, whereas mainly in cytoplasm when expressed at low level (Figure 3 and 4). The results demonstrated that the differential distribution of the fluorescence fusion proteins was associated with their expression level in Sl-HP cells.

**Figure 6:**
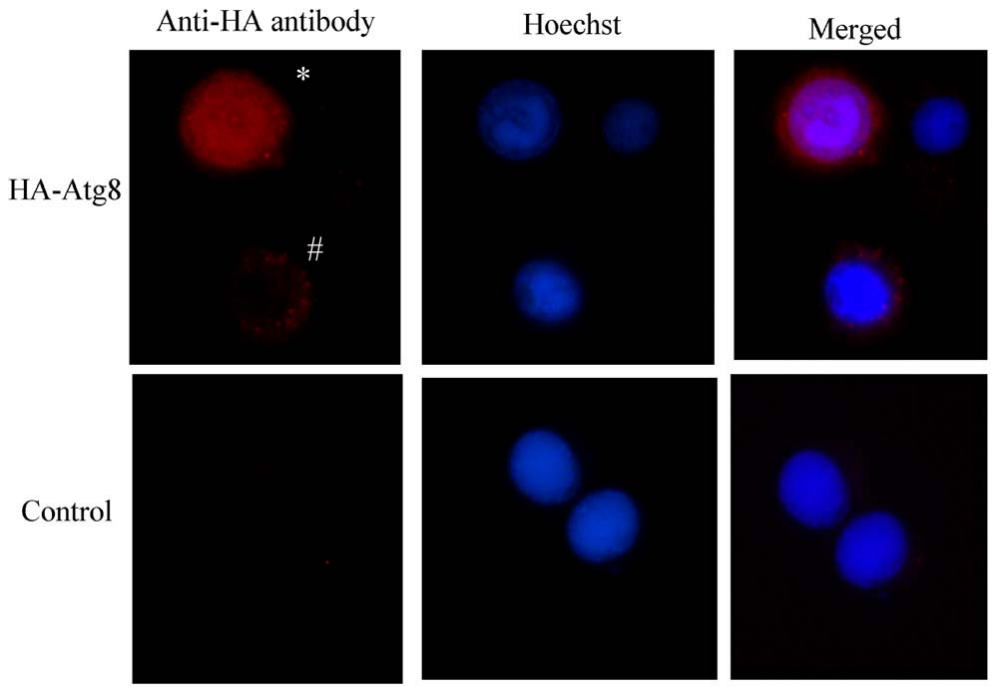
The distributions of HA-Atg8 between cytoplasm and nucleoplasm when HA-Atg8 was expressed at high level using baculovirus system. Sl-HP cells were infected with the recombinant baculovirus rBac-HA-Atg8 at moi of 0.2. The expressed HA-Atg8 was detected by immunofluorescence assay 32 h post-infection (upper panel). *: showing that HA-Atg8 expressed at high level is enriched in both cytoplasm and nucleoplasm; #: indicating HA-Atg8 expressed at moderate level is mainly located in cytoplasm. The control cells were infected with the recombinant baculovirus rBac-EGFP–actin expressing the EGFP-actin fusion protein under the control of polyhedrin promoter (down panel).

To assay whether the localization of the Atg8 protein without fluorescent protein tag was also associated with the expression level, a recombinant baculovirus (rBac-HA-Atg8) expressing HA-Atg8 under the control of polyhedrin promoter was constructed. As expected, different expression level of the recombinant protein was observed because of the low moi among individual cells using immunofluorescence staining. HA-Atg8 expressed at high level was located in both cytoplasm and nucleoplasm and that expressed at moderate level was mainly located in cytoplasm (Figure 6).

### The Cleavage of EGFP-Atg8 Differed from That of mCherry-Atg8

**Figure 7:**
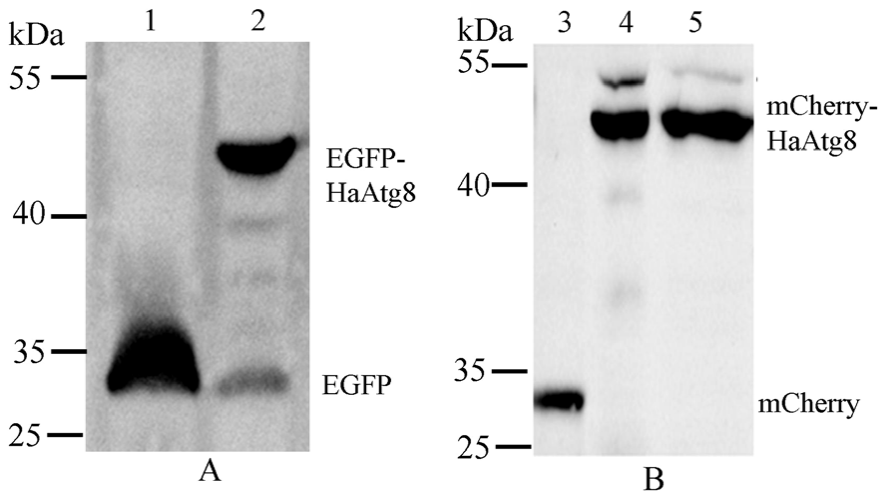
Comparison of the expressed EGFP-Atg8 and the expressed mCherry-Atg8 in Sl-HP cells. (A) EGFP-Atg8; (B) mCherry-Atg8. 1. EGFP; 2. EGFP-Atg8; 3. mCherry; 4. mCherry-Atg8 (total protein); 5. mCherry-Atg8 (nucleoplasm). Mouse anti-GFP monoclonal antibody and anti-mecherry antibody were used as primary antibodies for western blot assay in (A) and (B), respectively.

To investigate why the distribution of EGFP-Atg8 was different from that of mCherry-Atg8 even if both the plasmids (pEGFP-Atg8(EGFP) and pmCherry-Atg8(EGFP)) were similar (Figure 3 and 4), the expressed proteins were assayed using western blot. It revealed that the band profile of EGFP-Atg8 was different from that of mCherry-Atg8 in Sl-HP cells (Figure 7). For the cells expressing EGFP-Atg8, two molecules were observed, the large one corresponding to the full length of EGFP-Atg8 and the short corresponding to the EGFP which probably resulted in the cleavage of EGFP-Atg8 in lysosomes via the pathway of autophagy (Figure 7). For the cells expressing mCherry-Atg8, two bands with almost same molecular weight were detected (Figure 7). The low molecular weight protein of these two proteins was likely mCherry-Atg8 compared to EGFP-Atg8 on PVDF membrane (Figure 7). However, the reason for producing the high molecular weight protein in the cells expressing mCherry-Atg8 is unknown.

### Both Cleaved and Non-cleaved Atg8 Fusion Proteins Could Enter Nucleus

**Figure 8:**
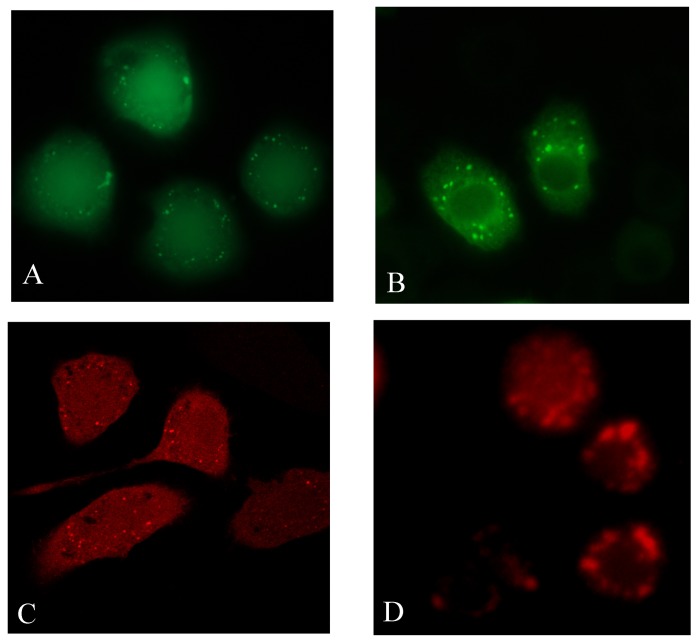
Comparison of the distribution of various fusion Atg8 and the influence of different FBS on the localization of mCherry-Atg8. (A) pie2/EGFP-Atg8; (B) pEGFP-Atg8(EGFP); (C) pmCherry-Atg8^G116^(EGFP); (D) pmCherry-Atg8(EGFP), showing that the mCherry-Atg8 was enriched in cytoplasm when FBS with the different lot number was used (also see Figure 4B).

**Figure 9:**
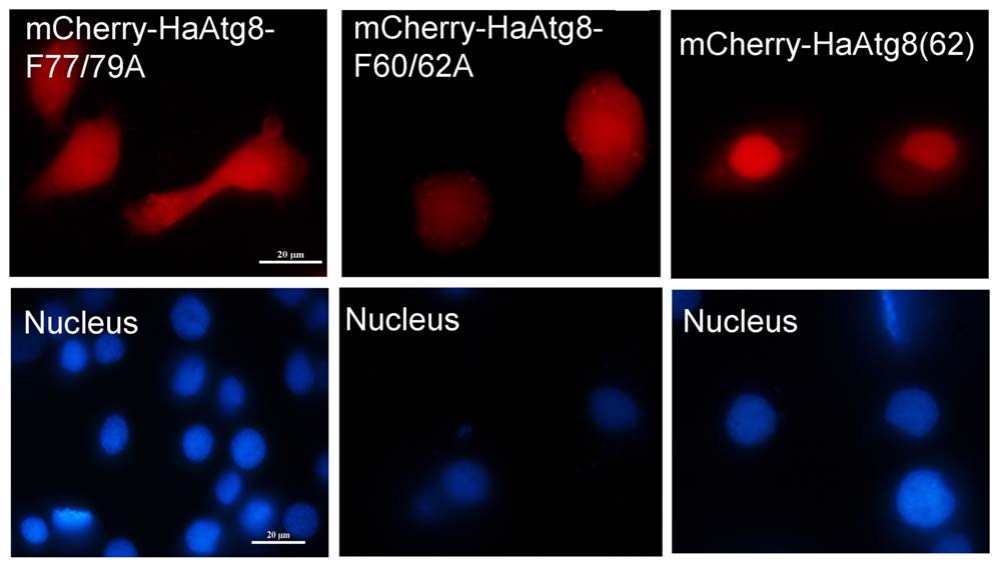
Subcellular localization of the mutant and truncated mCherry-Atg8 in Sl-HP cells. mCherry-HaAtg8^F77/79A^ lost the ability of forming punctual dots, compared to mCherry-HaAtg8^F60/62A^. The mCherry-Atg8^62^ truncated at C-terminus was enriched in nucleoplasm.

To investigate which of the cleaved and non-cleaved Atg8 fusion proteins mediated by Atg4 was/were distributed in nucleus, mCherry-Atg8^G^, mCherry-Atg8^F60/62A^ and mCherry-Atg8^F77/79A^ were expressed in Sl-HP cells. Although EGFP-Atg8 fusion protein expressed via pEGFP-Atg8(EGFP) was mainly accumulated in cytoplasm, and EGFP-Atg8 fusion protein expressed via pie2-EGFP-Atg8 was localized in both cytoplasm and nucleoplasm, both of them could form fluorescent punctual dots under normal condition and the number of the dots had no significant difference between the plasmids-transfected cells, suggesting that the fluorescent punctual dots, which might be autophagosomes, had no influence on the distribution of EGFP-Atg8 at some degree under normal condition (Figure 8A and B). To determine the localization of the Atg8 cleaved by Atg4, the truncated mutant mCherry-Atg8^G^ exposing glycine residue at the C termius was expressed in Sl-HP cells. The result of the fluorescence microscopy revealed that mCherry-Atg8^G^ exposing glycine residue was distributed in both cytoplasm and nucleus (Figure 8C). However, when autophagy was activated under some conditions such as the change of fetal bovine serum [24], the transportation of mCherry-Atg8 from nucleus to cytoplasm occurred (Figure 8D). In addition, fluorescence microscope revealed that the mutant mCherry-Atg8^F77/79A^ lost the ability of forming autophagosomes, but the mutant mCherry-Atg8^F60/62A^ did not (Figure 9). This is consistent with the report that Atg4 might not recognize Atg8^ F77/79A^ [24] and cleave it. Both mCherry-Atg8^F77/79A^ and mCherry-Atg8^F60/62A^ could be located in cytoplasm and nucleopolasm no matter they were cleaved by Atg4 or not (Figure 9), suggesting that the distribution of Atg8 fusion proteins in cytoplasm and nucleoplasm was not due to the cleavage of Atg8 mediated by Atg4. Our data showed that both the non-cleaved and cleaved mCherry-Atg8 was located in both cytoplasm and nucleoplasm and the truncated mCherry-Atg8^62^ (deletion of the amino acid residues after the 62^th^ amino acid residue of Atg8) was mainly accumulated in nuleoplasm (Figure 9).

### The Ratio of HA-Atg8-PE to HA-Atg8 Was Less Than That of Endogenous Atg8-PE to Atg8

**Figure 10:**
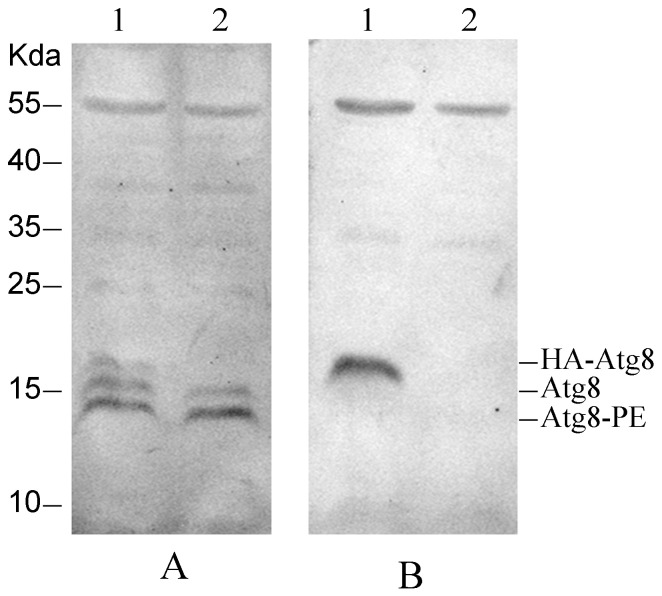
Processing of HA-Atg8 in the transfected Sl-HP cells. (A) Western blot assay using mouse anti-Atg8 serum; (B) Western blot assay using mouse anti-HA antibody. Lane1. The cells transfected with plasmid pHA-Atg8(EGFP); Lane 2. The cells transfected with pEGFP (Control).

To determine whether HA-Atg8 was processed to produce HA-Atg8-PE in Sl-HP cells, the expressed HA-Atg8 was analyzed using Western blot. Two bands for endogenous Atg8 and Atg8-PE in the transfected cells expressing HA-Atg8 were observed (Figure 10A). There was only one band for HA-Atg8 and no band for HA-Atg8-PE (Figure 10B). The result suggested that HA tag might have influence on lipidation of HA-Atg8 or the activities of enzymes for the lipidation were not enough for the processing of the over-expressed HA-Atg8.

In conclusion, the abundance of Atg8 has significant influences on its localization between cytoplasm and nucleoplasm, the cleavage of EGFP-Atg8 is different from that of mCherry-Atg8, and the transportation of the Atg8 fusion proteins from nucleus to cytoplasm is related to the increase of autophagy.

## Discussion

### The Proteins Transiently Binding to Atg8 in Cytoplasm Possibly Blocked the Transportation of Atg8 from Cytoplasm to Nucleoplasm when Atg8 Was Expressed at Low Level

A few of proteins binding to Atg8 have been reported to distribute in cytoplasm [25]–[27]. From the data in the present study, we propose that the binding of the cellular proteins to Atg8 forms large complex to inhibit the entry of Atg8 complex into nucleus. In our present experiments, the high level expression of fluorescence-tagged Atg8 might result in the production of free fluorescence-tagged Atg8, and no more binding proteins of Atg8 could bind to the over-expressed fluorescence-tagged Atg8. Thus, the free fluorescence-tagged Atg8 was passively transported into nucleoplasm.

### The C Terminus of Atg8 Is Critical for the Localization of Atg8 in Cytoplasm

The mutant of Atg8^G116A^ was enriched in nucleoplasm rather than in cytoplasm, suggesting that the glycine^116^ of Atg8 might be critical for the distribution of Atg8 in cytoplasm [23]. The mCherry-truncated Atg8^62^ in which the C terminus (62-117) was removed significantly increased in nucleoplasm (Figure 8). These results suggest that the binding domain of Atg8 to cytoplasmic proteins might be located in the C-terminus of Atg8.

### F77/79 Residues of Atg8 Is Essential for Autophagy

The crystal structure of microtubule-associated protein light chain 3 was determined [28]. Amar et al. has recently identified two new sites in the ubiquitin-like protein Atg8 which are essential for autophagy [29]. For Lepidopteran insect Atg8, the mutant mCherry-Atg8^F60/62A^, but not the mutant mCherry-Atg8^F77/79A^ formed autophagosomes (Figure 9), indicating that the residues in the position of F77/79 are essential for the function of Atg8.

### The Cleavage of Fluorescence Protein Tagged Atg8

The cleavage profile of mCherry-Atg8 was different from that of EGFP-Atg8 in Sl-HP cells. There were two major bands with the molecular weights of 43 and 27 kDa for the expressed EGFP-Atg8 (Figure 5). However, there were two bands for the expressed mCherry-Atg8 with molecular weights about 45 and 50 kDa, respectively, and no cleavage was detected for mCherry (Figure 5).The 50 kDa protein might be a complex formed by the full length of mCherry-Atg8 protein (about 45 kDa) and other cellular component to result in delaying its migration on SDS-PAGE gel. Compared to EGFP-Atg8, no significant cleaved mCherry-Atg8 was detected. Some studies showed that the abundance of free EGFP fragments resulting from the cleavage of EGFP-Atg8 in autolysosomes might indicate the increase of autophagic flux [30], [31]. Considering that the cleaved EGFP-Atg8 resulted from the digestion of lysosomes via the pathway of autophagy and no cleaved mCherry was detected (Figure 7), the result suggested that the involvement of mCherry-Atg8 in autophagy might not be as active as that of EGFP-Atg8.

### The Possible Mechanism for the Differential Expression Levels of EGFP-Atg8

For pEGFP-Atg8(EGFP), the mechanism for the moderate level of EGFP-Atg8 expression is that the CMV promoter could compete with the ie2 promoter for the recruitment of basic transcription accessory proteins even though the CMV promoter is not active in insect cells. The EGFP-Atg8 was expressed at high level in the pie2/EGFP-Atg8 construct because the CMV promoter was deleted.

### The Physiological Function of Atg8 Shuttling between Cytoplasm and Nucleoplasm

A few proteins interacting with Atg8 have been reported to have multiple functions [25]–[27]. To date, no nucleoplasmic protein has been identified to interact with Atg8. The shuttling of Atg8 between cytoplasm and nucleoplasm might regulate the functions of the interacting proteins in cytoplasm. In the present study, we demonstrated the shuttling of mCherry-Atg8 from nucleus to cytoplasm (Figure 8D). The shuttling was beneficial for mCherry-Atg8 to form autophagosomes when autophagy was activated, suggesting that the distribution (storage) of Atg8 in nucleus could rapidly regulate autophagy under some conditions. The expression of Atg8 rapidly increased before the appearance of pupation in insects [24], [32], [33]. The shuttling of Atg8 between nucleus and cytoplasm might exist in some cells at the stage of pre-pupation, which might play important physiological roles during the development of insect.
